# Protocol for repeated *Plasmodium chabaudi* infection in a murine malaria model

**DOI:** 10.1016/j.xpro.2026.104850

**Published:** 2026-09-21

**Authors:** Federica Bruno, Jean Langhorne, Cristina Lo Celso

**Affiliations:** 1 Department of Life Sciences, Imperial College London, London SW10 9NH, UK; 2 Francis Crick Institute, London NW1 1AT, UK; 3 Centre for Haematology, Department of Immunology and Inflammation, Imperial College London, London W12 0NN, UK

**Keywords:** Immunology, Stem Cells, Special Issue, NMGN Focused Collection

## Abstract

In malaria-endemic regions, individuals experience repeated malaria infection throughout life. Here, we present a protocol to investigate the immunological mechanisms triggered by repeated exposure using a murine model of sequential infections with the rodent parasite *Plasmodium chabaudi chabaudi*. We provide a detailed and standardized series of steps covering primary infection, natural recovery without treatment, and subsequent secondary infection. This approach offers a robust framework to investigate the effects and long-term consequences of repeated malaria infections on the host.

For complete details on use and execution of this protocol, please refer to Stephen et al.,^1^ Pérez-Mazliah et al.,^2^ and Achtman et al.^3^

## Highlights

• Standardized protocol for repeated *Plasmodium chabaudi* infection in mice
• Natural parasite clearance enabling re-infection without antimalarial treatment
• Investigation of immunity and long-term host responses to repeated malaria

## Abstract (editor highlights)

Publisher’s note: Undertaking any experimental protocol requires adherence to local institutional guidelines for laboratory safety and ethics.

## Graphical abstract

**Figure:**
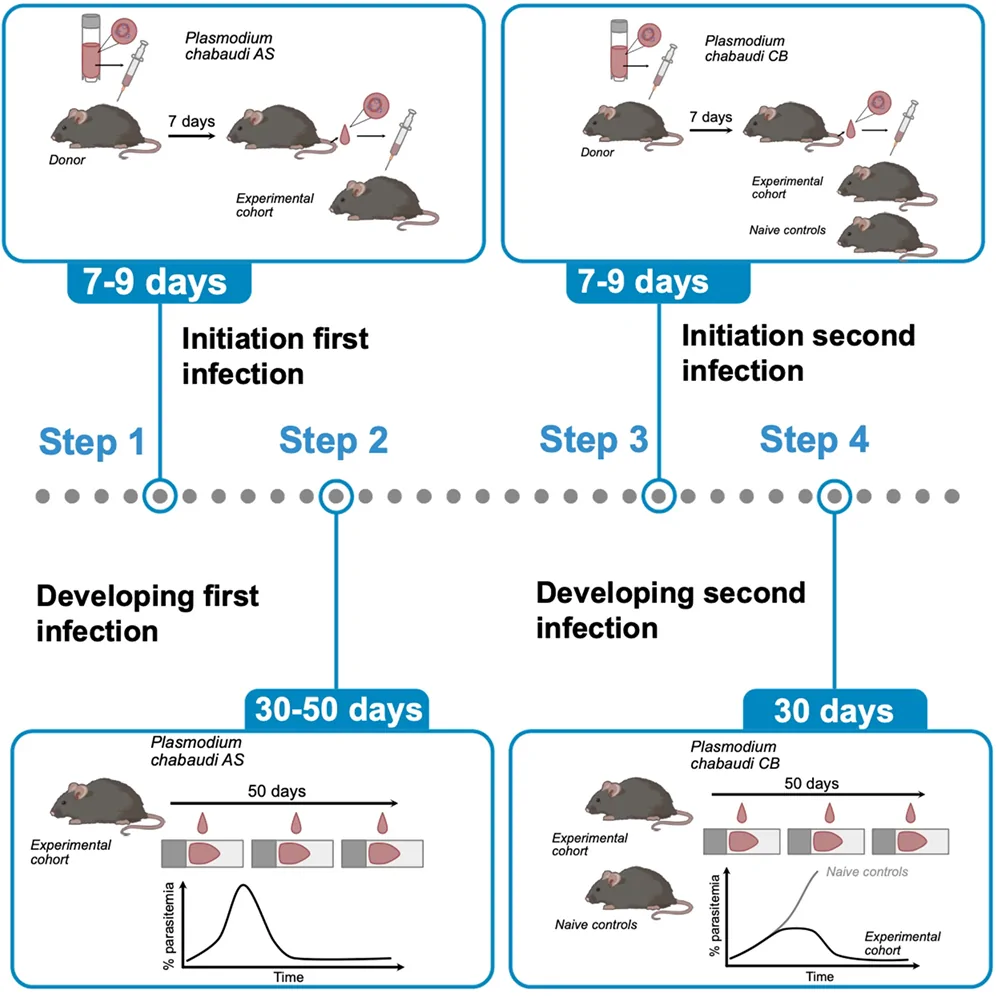

## Before you begin

Malaria, caused by the *Plasmodium* parasite, is a severe and life-threatening disease and it is still a global cause of mortality. Importantly, due to the polymorphism of *Plasmodium*, individuals living in endemic areas are often re-infected by antigenically different parasites which evade the immune response.^4^ Recently, it was demonstrated that individuals contracting malaria several times throughout their lifetime are protected against clinical disease. Importantly, an increasing body of evidence underscores how protective immunity develops after one or two episodes of malaria, resulting in a reduced risk of severe disease and control of parasitemia at low levels.^4^^,^^5^^,^^6^^,^^7^ Moreover, this partial immunity has been shown to remodel the host immune response to prevent acute systemic activation, highlighting how the host is able to tolerate the parasite. While clinical immunity to malaria has been historically thought to be beneficial as it prevents severe disease, recently adverse outcomes have come to light. Specifically, it has been associated with maternal and neonatal mortality and a higher risk of co-infections with other pathogens due to immunosuppression.^6^ Incomplete development of clinical immunity has been shown to contribute to long-term hidden pathologies such as chronic anemia, cognitive impairment and bone loss.^8^^,^^9^^,^^10^^,^^11^ Additionally, asymptomatic persistent malaria increases the transmission of the parasite and promotes the spread of drug-resistant strains. Importantly, while some level of exposure may provide immunity, ongoing malaria infections are a public health challenge due to their high costs in terms of human health and societal impact. The precise process beyond the establishment of clinical immunity against malaria is still unclear, and experimental models of repeated infection are ideal to investigate the mechanisms underlying this phenomenon. We established a murine model of repeated malaria whereby following recovery from first infection, mice undergo a second malaria episode. While we mainly use this model to study the hematopoietic response in repeated malaria, it can be readily applied to a broad range of research questions, including the investigation of immune responses, parasite-targeted interventions, and rescue of long-term effects.

### Innovation

Most models of repeated murine malaria infection use *Plasmodium* strains with high severity and risk of death, requiring anti-malaria treatments to clear the parasite before initiating a second infection.^12^^,^^13^^,^^14^ By utilizing a self-recovery model of *Plasmodium chabaudi chabaudi* infection we have detailed a protocol whereby the immune system of mice is able to clear the parasite naturally followed by a second infection. This approach enables the study of biological systems without the confounding effects of pharmacological interventions, thereby more accurately reflecting real-world conditions, where re-infection is often associated with asymptomatic malaria that typically remains untreated.

### Institutional permissions

All animal work, including animal care and experimental procedures, was carried out at Imperial College London (ICL) in accordance with the current UK Home Office regulations (ASPA 1986) under license number PP9504146. All work should be carried out as containment level 1 (CL1).

### Storage of frozen parasite

1. Acquire stabilate aliquots (frozen stock of infected blood) of *Plasmodium chabaudi chabaudi* (Strains: AS and CB) from the European Malaria Reagent Repository, University of Edinburgh.
2. Maintain the frozen stock in liquid nitrogen.

## Key resources table

**Table:** 

REAGENT or RESOURCE	SOURCE	IDENTIFIER
**Experimental models: Organisms/strains**		

Mouse: C57BL/6	Charles River	Strain: 027

**Biological samples**		

Frozen stock of blood infected with *Plasmodium chabaudi chabaudi* AS	European Malaria Reagent Repository, University of Edinburgh https://www.malariaresearch.eu/	*Plasmodium chabaudi chabaudi* AS
Frozen stock of blood infected with *Plasmodium chabaudi chabaudi* CB	European Malaria Reagent Repository, University of Edinburgh https://www.malariaresearch.eu/	*Plasmodium chabaudi chabaudi* CB

**Chemicals, peptides, and recombinant proteins**		

Saline (0.9% sodium chloride)	Vetivex 1	Cat#QB05BB01
Heparin Sodium (1000 IU/mL)	Wockhardt	Cat#FP1077
Giemsa stain, modified solution	Sigma-Aldrich	Cat#48900-500ML-F
Methanol	Sigma-Aldrich	Cat#34860

**Other**		

Microscope slides	VWR	Cat#631-1554

## Step-by-step method details

### Initiation of first infection

**Timing: 7–9 days**

In this step, a first malaria infection is induced with *Plasmodium chabaudi* AS in an experimental cohort of naïve mice. This requires blood from one infected donor mouse. This can be either by pre-infecting a mouse with frozen stabilate (step 1) or by blood passage from a mouse in another experimental cohort (skip to step 3).

**CRITICAL:** The majority of mice survive *Plasmodium chabaudi* AS infection without any major clinical signs, however rarely some mice might show signs of disease during the acute phase (day 9 to 15, or parasitaemia > 10%) of the primary infection, such as weight loss, blood in urine and pallor of the paws consistent with anaemia. From day 5 post-infection onwards, mice need to be monitored regularly for body weight, parasitaemia, and clinical signs to assess disease severity. Humane endpoints include the development of a hunched posture, reduced mobility, decreased strength, lethargy, or rapid weight loss exceeding 10% within 24 h or 15% over 48 h. Animals reaching any of these endpoints will be humanely euthanised. The likelihood of severe disease and mortality varies depending on the *P. chabaudi* strain used, as well as the age and sex of the host. The *P. chabaudi* CB strain is more virulent than the AS strain and is associated with much increased disease severity and reduced survival. We therefore recommend using the CB strain only after an initial AS infection is cleared. In addition, male and older mice have a slightly higher risk of developing severe infection. Throughout this protocol we use young (10–12 weeks) C57BL/6 female mice unless clearly stated otherwise.

***Note:*** Blood from one donor is sufficient to infect an experimental cohort of up to 50 mice.

**CRITICAL:** It is recommended that no more than 4 serial passages between mice are carried out before reverting to the frozen stabilate because prolonged and repeated serial- blood passage might select for increasingly virulent parasites.

1. Initiate infection in two donor mice 7–8 days prior infection of experimental cohort.
  a. Collect frozen stabilate of blood infected with *Plasmodium chabaudi* AS from liquid nitrogen.
  b. Transport the vial on dry ice to the mouse holding room.
  c. When ready to inject, rapidly thaw and mix the blood by rotating the vial between the palms of your hands.
  d. As soon as thawed, inject two donor mice with 100 μL of stabilate intraperitoneal (IP).

***Note:*** Both immunocompetent (C57BL/6) or immunosuppressed mice (such RAG1 KO) can be used as donors; when using immunocompetent mice parasitemia growth curve needs to be monitored as they will clear the infection quickly. Donor mice need to be at least 6 weeks of age and can be male or female.

2. Throughout the infection, monitor parasitemia by thin blood films (blood smears) every 2–3 days.
  ***Note:*** While light microscopy of Giemsa-stained blood smears remains the predominant and WHO-recommended standard for malaria parasitemia assessment worldwide, recently flow cytometry has been proposed to increase throughput.^15^
  a. Collect a drop of blood (5 μL) by tail vein from the infected mice.
  b. Drop the blood close to one narrow edge of a microscope slide.
  c. Use a second slide at a 45° angle to drag the blood first slightly backward and then all the way forward in a steady motion. This forms a monolayer of cells (Figure 1).

**Figure 1:**
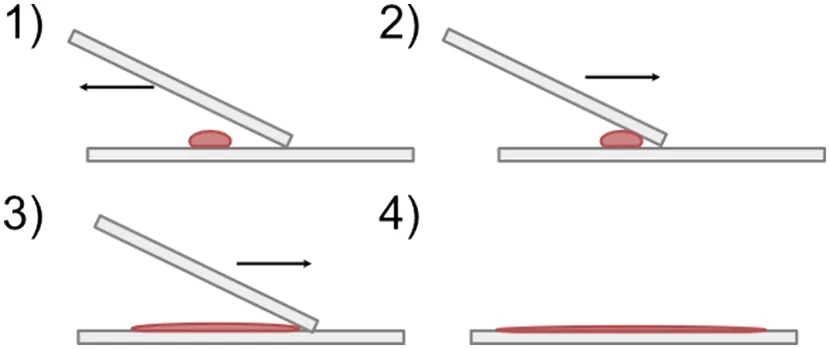
Thin blood film preparation Collect 5 μL of blood from the tail vein of infected mice, place it at one end of a microscope slide, and use a second slide held at a 45° angle to spread the blood into a thin monolayer.
  d. Gently squeeze 99% methanol on the slide to fix the blood.
  e. Let the slides air dry for 10 minutes.
  f. Meanwhile, prepare the Giemsa staining solution by diluting Giemsa solution 1 in 5 with distilled water.
  g. Incubate the slides in the Giemsa staining solution for 20 minutes at room temperature.
  h. At the end of the incubation, wash the slides with distilled water followed by air drying.
  i. Once dry, visualize the slides with a light microscope equipped with a ×100 oil immersion objective.
  j. Use a grid in the eyepiece to identify a field containing around 200 red blood cells (RBCs).
  k. Count the number of RBCs and infected RBCs (iRBCs) (Figure 2) contained in the field and use the following formula to calculate parasitemia:
    $$parasitemia\;\left(\%\right)=\frac{number\;of\;iRBCs\times 100}{number\;of\;RBCs}$$

**Figure 2:**
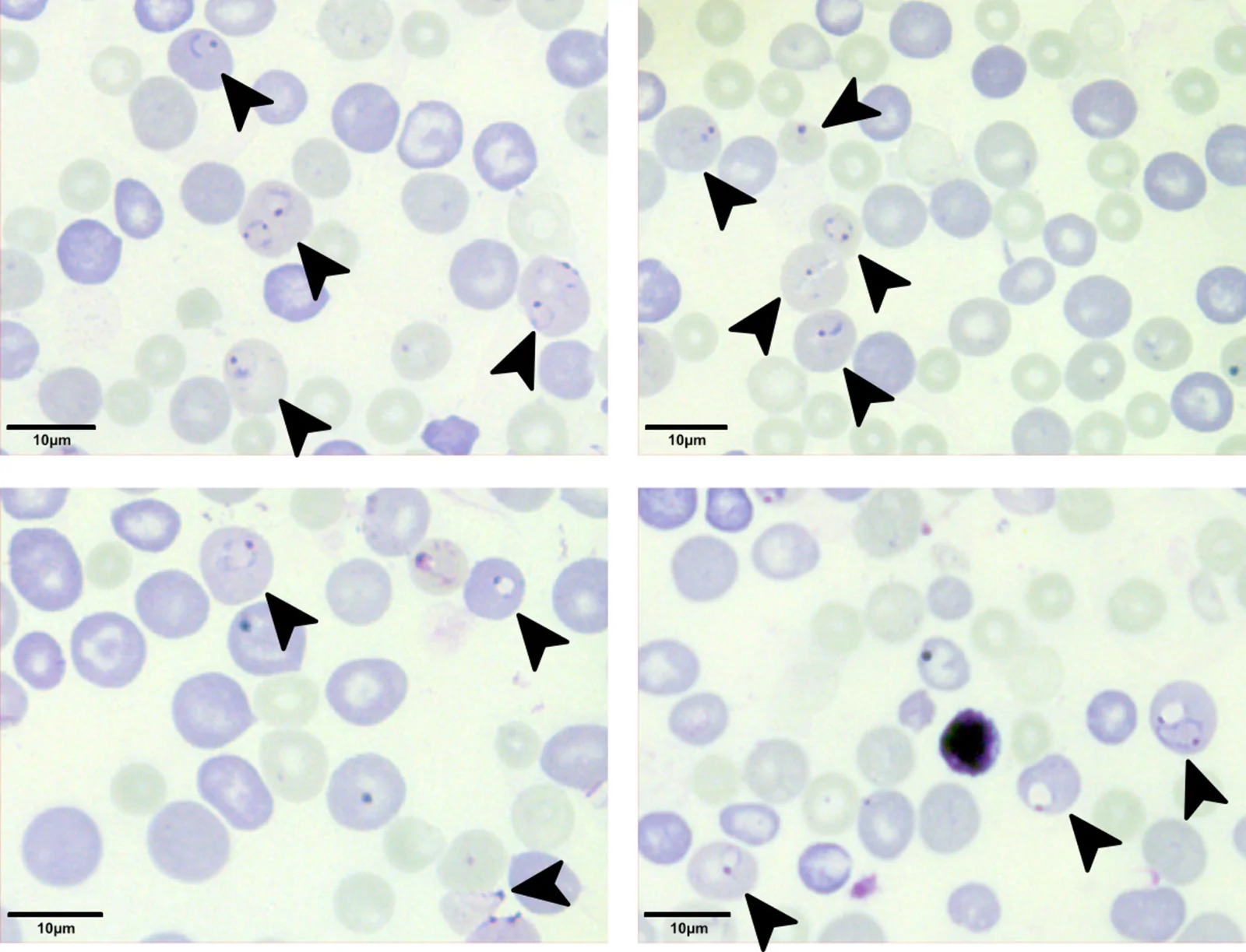
Identification of RBCs and iRBCs Representative Giemsa-stained thin blood film showing RBCs and iRBCs (arrowheads). Scale bar = 10 μm.
  l. Calculate the final parasitemia by averaging the results of 3 fields minimum.
    ***Note:*** When parasitemia is < 1%, assess twenty to thirty fields.
3. At day 7 post infection, determine parasitemia in donor mice as described in step 2.

**CRITICAL:** Donor parasitemia should be at least 2% to initiate infection. Two donors are infected to allow selection of a donor with adequate parasitemia.

4. Calculate blood dilution factor based on the donor parasitemia to prepare an inoculum containing 1 × 10^6^ iRBCs per 100 μL injection volume.
5. Prepare an aliquot of diluted heparin (25 U/mL) and an aliquot of saline.

***Note:*** Aliquots of heparin and saline should be prepared fresh every 2–3 weeks.

6. Place donor mouse in a heath box for 10 minutes to be able to collect enough blood.
7. Collect 10 μL of blood by the tail vein into 30 μL of diluted heparin.
8. Dilute the collected blood into saline based on dilution factor previously calculated.
9. Inject recipient mice with 100 μL containing 1 × 10^6^ iRBCs.

**CRITICAL:** Collect and inject blood as rapidly as possible to preserve parasite’s viability.

10. Once all experimental mice are injected, cull the donor mice with a SK1 method.

### Monitor first infection and determine recovery

**Timing: 30–50 days**

In this step, monitor first infection by measuring mouse weight and parasitemia to determine the different phases of infection. We take undetectable parasitemia as the criterion to define cleared infection.

11. Carry out blood smear from experimental cohort every 2–3 days in order to determine parasite growth curve.
  a. Once that the infection model is established and parasitemia growth curve is reliable, blood smear can be done less frequently.
12. Employ parasite growth curve to determine acute and recovery phases and to choose timepoints of interest.

***Note:*** When using *Plasmodium chabaudi* AS, parasitemia is expected to peak at approximately 20%–30% between day 9 and day 12 post-infection and to become undetectable in the blood by approximately day 30 post infection. Peak weight loss typically occurs during the same period of peak parasitemia; however, this is not consistently observed across all mice.

### Initiate and monitor second infection

**Timing: 30 days**

In this step, the second malaria infection is initiated in recovered mice with a different strain of parasite from the primary infection: e.g., primary infection with *Plasmodium chabaudi* AS and second infection with *Plasmodium chabaudi* CB.

**CRITICAL:** In this model, the more virulent *Plasmodium chabaudi* CB strain is used only as the secondary challenge infection. Primary infection with *P. chabaudi* CB, compared with the less virulent AS strain, results in more severe disease, characterised by greater weight loss and higher parasitaemia. Animals are monitored closely throughout the study and will be humanely euthanised if predefined humane endpoints are reached, including hunched posture, reduced mobility, decreased strength, lethargy, or rapid weight loss. The use of the less virulent AS strain for primary infection reduces the risk of animals developing severe disease and reaching humane endpoints, while still allowing investigation of the immune response to subsequent challenge with the more virulent CB strain.

13. At day 53 post AS infection, infect two donor mice with *Plasmodium chabaudi* CB as described in Step 1.
14. At day 60 post AS infection, initiate second infection as described in Step 3–10.
15. Determine phase of infection by monitoring mice weight and parasitemia as described in Step 13–14.

**CRITICAL:** Infect some naïve mice to confirm that infection develops as expected; mice infected with *Plasmodium chabaudi* CB as a first infection are anticipated to develop severe infection and should be culled with a SK1 method once they reach 40% parasitaemia, approximately day 11 post-infection.

## Expected outcomes

This protocol allows the establishment of a repeated murine malaria model. This involves two malaria episodes, where each is characterized by an acute infection and a recovery phase. Specifically, during first infection, parasites are detectable in the blood as soon as day 5 post infection, although at low levels (<3%). Parasitemia is expected to peak to approximately 20%–25% between day 9 to day 11 post infection followed by a gradual decrease (Figures 3A and 3B). Peak infection is associated with some transient weight loss, although this is not consistenly observed in all mice (Figures 3C and 3D). As a result, we defined the window of acute infection from day 9 to day 15 post infection, when parasitemia is >10%. By day 30 post infection, the parasite is not detectable anymore in the blood (Figures 3A).

**Figure 3:**
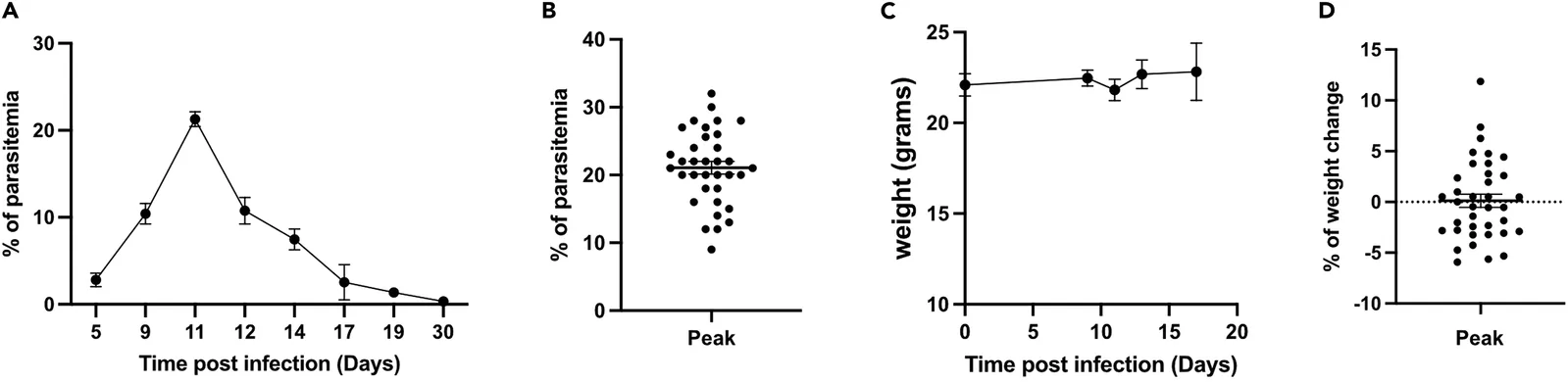
Infection dynamics of *Plasmodium chabaudi* AS (A) Parasitemia levels (% of iRBCs) in mice throughout *P. chabaudi* AS infection. (B) Parasitemia at peak infection of *P. chabaudi* AS. (C) Body weight of mice throughout *P. chabaudi* AS infection. (D) Body weight change at peak infection of *P. chabaudi* AS. In A–D *n* = 35 infected mice pooled from 5 independent experiments. In B and D each dot represents an animal. Data are represented as mean ± SEM.

Importantly, when using *Plasmodium chabaudi* AS, the acute phase of infection highly depends on the sex and age of the mice. We demonstrated that male mice exhibit higher parasitemia at peak infection (>25%) compared to female mice, which is associated with greater weight loss (Figures 4A–4C). Similarly, infection of older mice (65 weeks of age) resulted in higher parasitemia and greater weight loss compared to younger mice (10 weeks of age) (Figures 4D and 4E). Notably, older mice also exhibited reduced survival, highlighting the need for close monitoring when infecting aged animals (Figure 4F).

**Figure 4:**
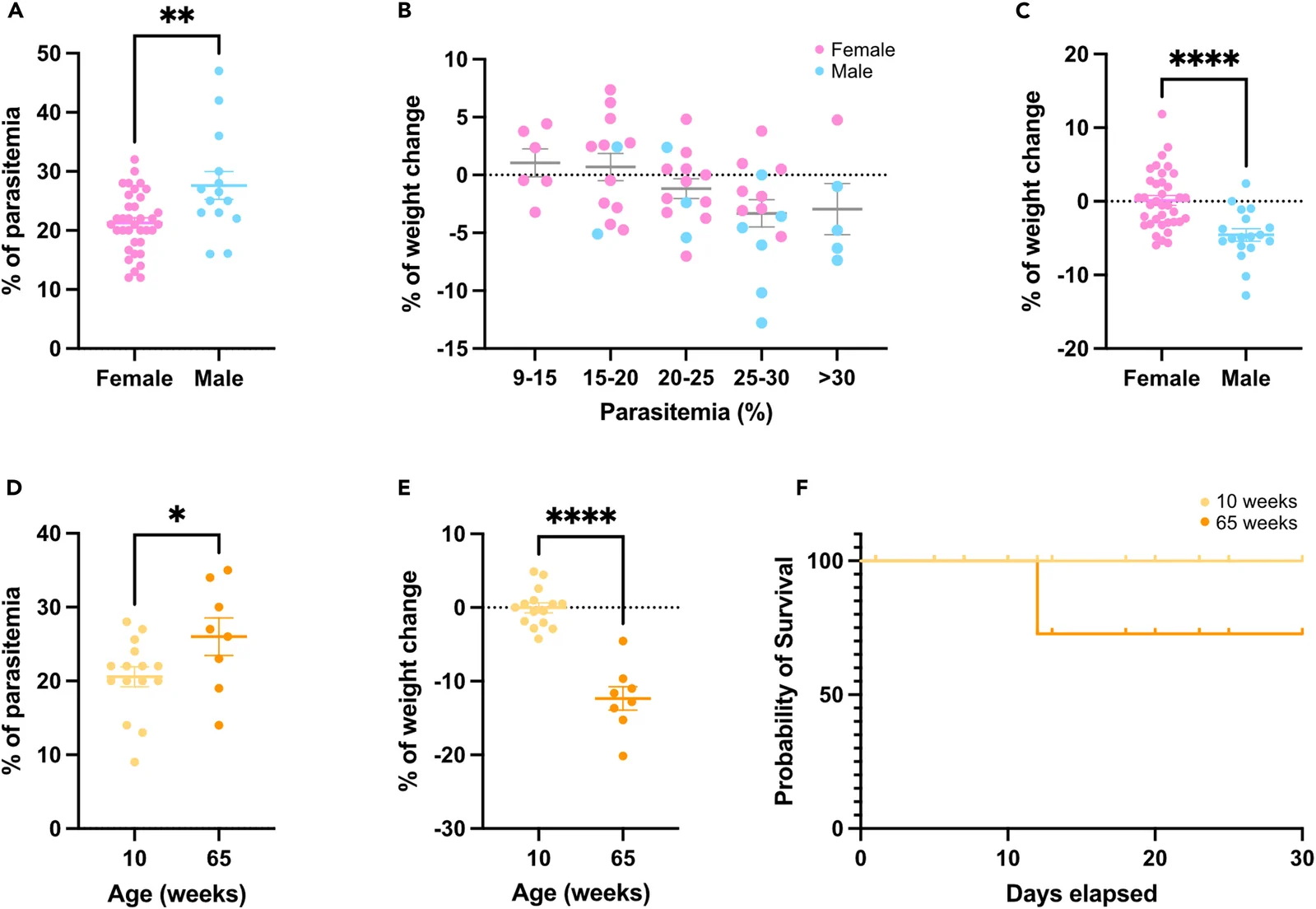
Comparison of *Plasmodium chabaudi* AS infection dynamics between female and male mice and between young and old mice (A) Parasitemia in male and female mice at peak infection of *P. chabaudi* AS. (B) Body weight changes in male and female mice with different levels of parasitemia at peak infection with *P. chabaudi* AS. (C) Body weight change in male and female mice at peak infection of *P. chabaudi* AS. (D) Parasitemia in old (65 weeks) and young (10 weeks) mice at peak infection of *P. chabaudi* AS. (E) Body weight changes in old and young mice throughout *P. chabaudi* AS infection. (F) Probability of survival of old and young mice during *P. chabaudi* AS infection. In A–E each dot represents an animal. In A–C *n* = 35 and 14 for female and male infected mice pooled from 5 independent experiments. In (D–F) *n* = 15 and 8 for young and old infected mice pooled from 3 independent experiments. Data are represented as mean ± SEM. Statistically significant differences were determined using Student’s *t* test (∗∗∗∗p≤ 0.0001; ∗∗∗ p≤0.001; ∗∗ p≤0.01; ∗ p≤0.05).

In addition, the course of infection varies between *Plasmodium chabaudi* strains.^16^^,^^17^ By comparing, *Plasmodium chabaudi* AS and *Plasmodium chabaudi* CB we demonstrated that the latter is associated with more severe disease, characterized by higher parastiemia levels (approximately 40%), greater body weight loss and markedly reduced survival (Figures 5A–5C). Collectively, these factors should be taken into account when initiating a new infection, both to ensure appropriate animal monitoring and to determine suitable experimental timepoints.

**Figure 5:**
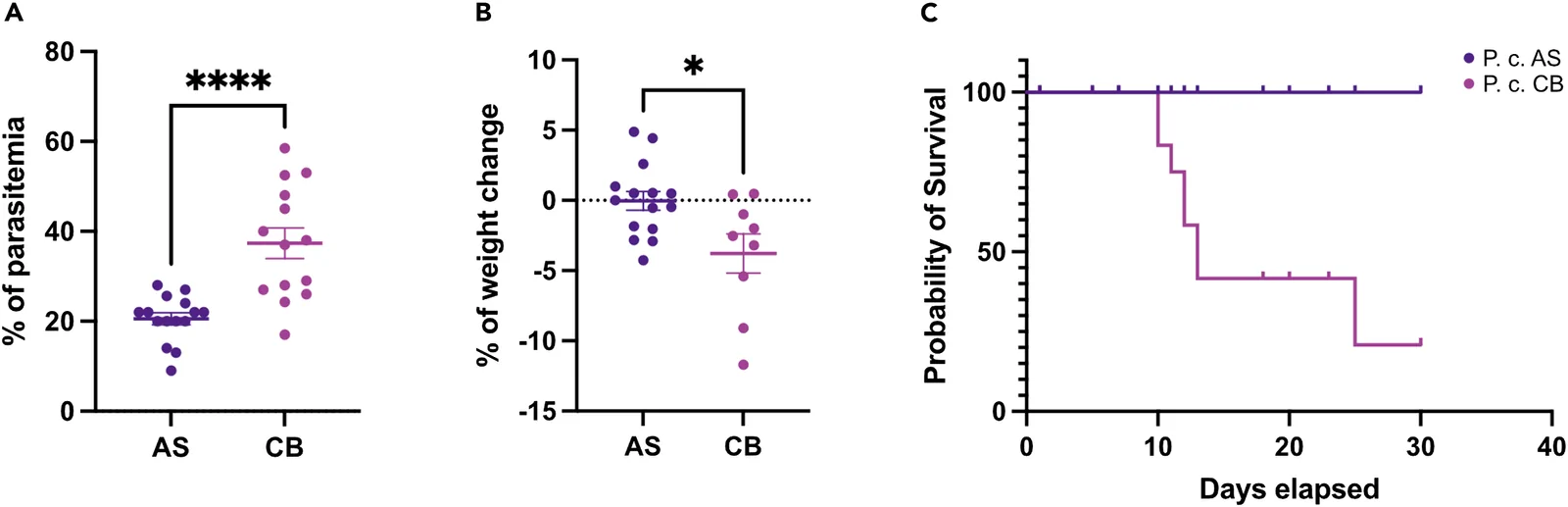
Infection dynamics of *Plasmodium chabaudi* AS vs *Plasmodium chabaudi* CB (A) Parasitemia in mice at peak infection with *P. chabaudi* AS or CB. (B) Body weight change at peak infection of *P. chabaudi* AS or CB. (C) Probability of survival during *P. chabaudi* AS or CB infection. In A and B each dot represents an animal. In A–C *n* = 15 and 9 for *P. chabaudi* AS and CB infected mice pooled from 2 independent experiments. Data are represented as mean ± SEM. Statistically significant differences were determined using Student’s *t* test (∗∗∗∗p≤ 0.0001; ∗∗∗ p≤0.001; ∗∗ p≤0.01; ∗ p≤0.05).

With regards to second infection, the timing of re-infection should be determined based on the recovery of the biological system under investigation. Because we employed this model to investigate hematopoietic system in response to infection, different hematopoeitc populations were assessed during first infection using phenotypic and proliferation assays, which indicated recovery of the system by day 60 post infection.^16^ As a result, a second infection was initiated at day 60 post infection with *Plasmodium chabaudi* CB. As expected, naïve mice experienced high parasitemia, marked body weight loss and reduced survival (Figures 6A and 6B). In contrast, mice that had recovered from the first infection displayed a markedly reduced parasitemia burden (at around 1%) from day 7 to day 14 post infection and with parasite becoming undetectable from day 17 onwards until the end of infection (Figures 6A and 6B).

**Figure 6:**
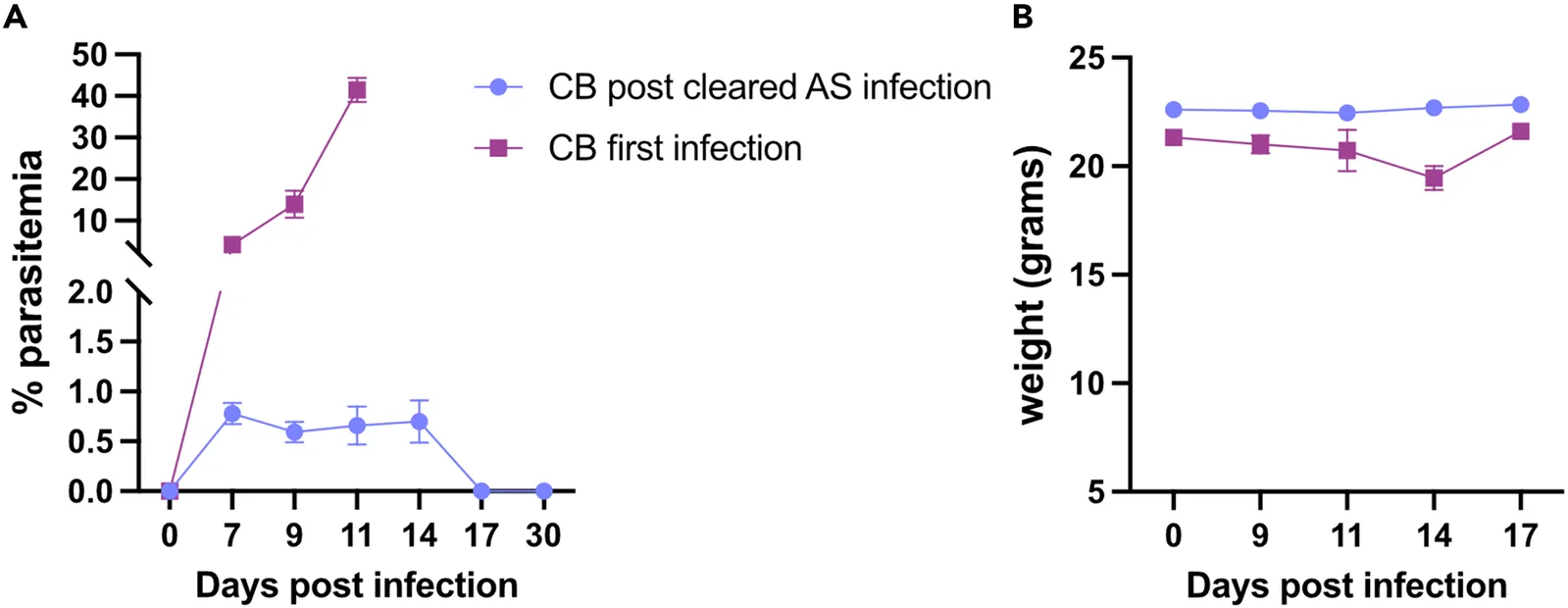
Infection dynamics of *Plasmodium chabaudi* CB in previously exposed or naïve mice (A) Parasitemia levels during *P. chabaudi* CB infection in naïve or mice that had previosly cleared *P. chabaudi* AS infection. Naïve mice were terminated at day 11 post infection due to the severity of disease. (B) Body weight during *P. chabaudi* CB infection of naïve or mice that had previosly cleared *P. chabaudi* AS infection. In A and B n = 9 and n = 30 for naïve or previously exposed mice pooled from 3 independent experiemnts. Data are represented as mean ± SEM.

## Limitations

One limitation of this protocol is variability in the timing of peak of first infection, which generally occurs within a 2–3-days span. Once the model is established, the parasitemia growth curve can be used to anticipate the peak and coordinate culling or sampling at the right time points. A further limitation of this protocol is the length of the experimental protocol, which is approximately 97 days. However, depending on the research question asked, the time between first and second injection could be adjusted. For instance, we determined that the hematopoietic system recovers at day 60 post first infection,^16^ and therefore to study the effects of a second infection on the haemtopoietic system, *P. chabaudi* CB could be administered at day 60. If the requirement to initiate the second infection is cleared parasitaemia, *P. chabaudi* CB could be administered as early as day 30 post first infection.^16^ Finally, AS and CB are not the only *Plasmodium chabaudi* strains available to the community. We selected them here because their combination as as follows by CB provides an effective and safe strategy to induce two subsequent waves of parasitemia in C57BL/6 mice. Other strains and strain combinations could be used instead, however the timing and intensity of each parasitemia wave could vary and would have to be carefully established before proceeding to analyse the response of the system or organ of interest.

## Troubleshooting

### Problem 1

Donor mice do not have adequate parasitemia to start an infection in experimental cohort.

### Potential solution

Allow one or two more days for the development of infection.

### Problem 2

Variability in infection development across mice.

### Potential solution

To account for potential variability in infection development, 1–2 additional mice should be infected to ensure sufficient numbers for statistical analysis. In the rare event that no parasites are detected in the blood by day 11 post-infection, those mice should be excluded from the experiment.

### Problem 3

The quality of the thin blood films is poor due to anemia.

### Potential solution

Always make sure that there is at least a drop of blood to smear, if required, heat the mice for 5 minutes at 37°. If no fields with 200 RBC can be found, count smaller fields containing 100 RBC but increase their number to a minimum of 6 fields. If the blood is very thin, then increase the angle of the second slide used to make the smear to more than 45°.

## Resource availability

### Lead contact

Further information should be directed to the lead contact, Cristina Lo Celso (c.lo-celso@imperial.ac.uk).

### Technical contact

Technical questions on executing this protocol should be directed to and will be answered by the technical contact, Federica Bruno (federica.bruno21@imperial.ac.uk).

### Materials availability

This study did not generate new unique materials.

### Data and code availability

This study did not analyze/generate datasets/code.

## Acknowledgments

C.L.C. was supported by the 10.13039/501100009053Wellcome Trust Investigator Award 212304/Z/18/Z and the 10.13039/501100000289CRUK Program Foundation Award C36195/A26770. F.B. is a recipient of an ICL DoLS PhD studentship. J.L. was funded by the core Francis Crick (FCI) program, supported by the Wellcome Trust, Cancer Research UK and the 10.13039/100014013UKRI 10.13039/501100000294Medical Research Council. We thank the Central Biomedical Services at Imperial College London for technical support.

## Author contributions

F.B. carried out the experiments, designed the project, and wrote and revised the manuscript. J.L. established the model of infection and revised the manuscript. C.L.C. designed the project and revised the manuscript.

## Declaration of interests

The authors declare no competing interests.
